# Runx3d controls the abundance and functional differentiation of CD4^+^CD8αα^+^ intraepithelial T cells

**DOI:** 10.1038/s41420-023-01415-z

**Published:** 2023-04-12

**Authors:** Can Li, Praveen Prakhar, Jung-Hyun Park

**Affiliations:** Experimental Immunology Branch, Center for Cancer Research, National Cancer Institute, National Institutes of Health, Bethesda, MD 20892 USA

**Keywords:** Immunology, Lymphocytes, Mucosal immunology, T cells

The small intestine (SI) epithelium harbors a peculiar population of αβ T cells that expresses both the CD4 and the CD8 coreceptor and thus appear as CD4 and CD8 double-positive (DP) cells [1]. Such DP intraepithelial lymphocyte (IEL) T cells differ from immature DP T cells in the thymus, as they are developmentally mature and functionally competent. SI IEL DP cells are also distinct from conventional CD4^+^ T cells as they have lost expression of the CD4-lineage specifying transcription factor ThPOK, and further differ from conventional CD8^+^ T cells as they express CD8αα homodimers instead of CD8αβ heterodimers as coreceptors [2].

The precise role of CD4^+^CD8αα^+^ DP T cells in gut immunity remains controversial and is a subject of active research [1]. DP IELs can exert immunostimulatory functions, as evidenced by the expression of effector molecules, such as granzyme B and IFNγ [1]. On the other hand, DP IELs are also proposed to exert immunosuppressive functions and to maintain immune quiescence in the gut [3]. Thus, the immunoregulatory role of CD4^+^CD8αα^+^ T cells are not clearly defined. The molecular mechanism by which CD4^+^CD8αα^+^ T cells are generated and maintained in the SI epithelium also remains unclear. We and others previously demonstrated a role of cytokines, chemokines, and other cell-extrinsic factors in promoting their development [4–6]. On the other hand, negative regulatory mechanisms that constrain DP IEL differentiation and maintenance have been mostly unknown. Here, we identify the runt-related transcriptional activator Runx3d as a key factor that limits the abundance but supports functional differentiation of CD4^+^CD8αα^+^ IELs, establishing a new regulatory circuitry of DP IEL differentiation and maintenance.

CD4^+^CD8αα^+^ T cells are highly enriched among IELs of the SI epithelium but not found in secondary lymphoid organs, such as lymph nodes (Fig. 1a). One of the major functions of CD4^+^CD8αα^+^ IELs is the production of the anti-inflammatory cytokine IL-10 [3], which we also confirmed in in vitro activated SI DP IELs (Fig. S1a). Consistent with an immunosuppressive role, we found that IL-2-deficient (*Il2*^–/–^) and IL-2 receptor β-deficient (*Il2rb*^–/–^) mice, which are models of gut inflammation, contained drastically reduced frequencies and numbers of DP IELs (Fig. S2a–d), suggesting an association of DP IELs with maintaining immune quiescence. Whether the loss of CD4^+^CD8αα^+^ IELs under conditions of gut inflammation is the cause or the consequence of autoimmunity is unclear and remains to be resolved. Nonetheless, the lack of CD4^+^CD8αα^+^ IELs in IL-2 signaling impaired mice was not due to an absence of Foxp3^+^ CD4 IELs, which are precursors of CD4^+^CD8αα^+^ IELs [7, 8], because they were not diminished in *Il2ra*^–/–^ mice compared to littermate controls (LMC) (Fig. S2e).

**Fig. 1:**
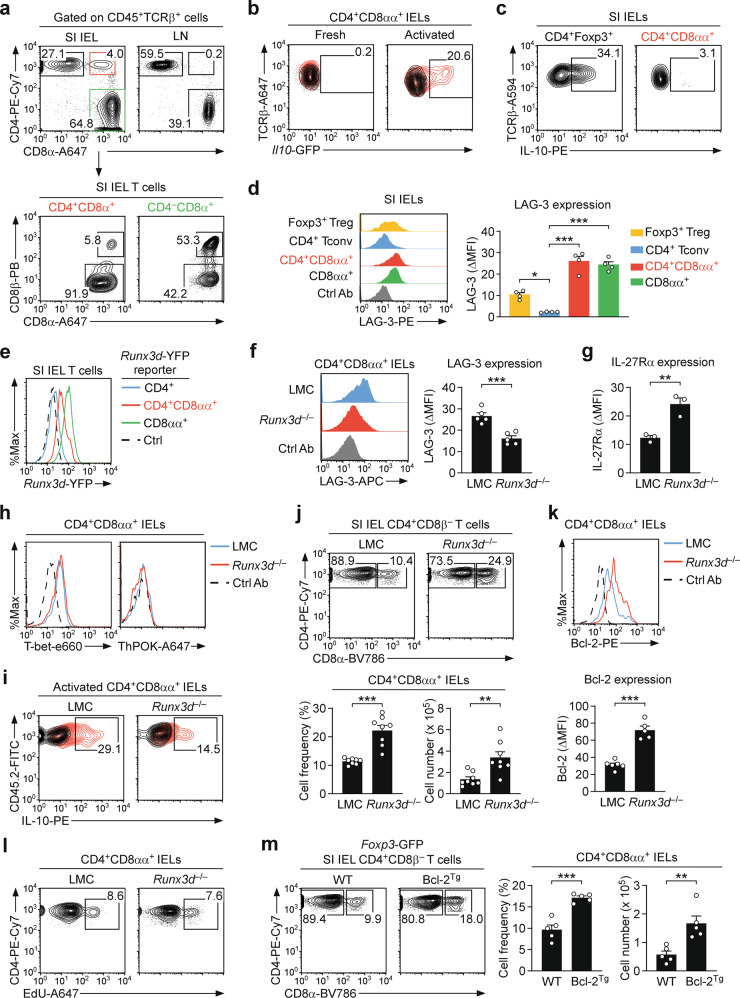
Runx3d controls CD4^+^CD8αα^+^ IELs in the SI epithelium. **a** CD4 versus CD8 profiles of αβ T cells in the SI epithelium and LN (top) and CD8α versus CD8β plot of CD4^+^CD8α^+^ DP and CD4^–^CD8α^+^ SI IEL T cells (bottom). The results are representative of 5 independent experiments. **b** GFP reporter expression after 4 h of PMA and ionomycin stimulation in freshly isolated and 3-day in vitro anti-CD3/CD28-activated CD4^+^CD8αα^+^ SI IEL T cells from WT (black line) and *Il10*-GFP reporter mice (red line). The results are representative of 3 independent experiments. **c** IL-10 expression after 4 h of PMA and ionomycin stimulation in freshly isolated CD4^+^CD8αα^+^ and CD4^+^Foxp3^+^ SI IELs. The results are representative of 3 independent experiments. **d** LAG-3 expression in the indicated αβ T-cell IEL populations of *Foxp3*-GFP reporter mice. The data are shown as histograms (left), and bar graphs are a summary of 2 independent experiments (right). **e** *Runx3d*-YFP reporter expression in the indicated populations of SI IELs. The results are representative of 3 independent experiments. **f** LAG-3 expression on *Runx3d*^–/–^ and littermate controls (LMC) DP IELs. The data are shown as histograms (left), and bar graphs are a summary of 3 independent experiments (right). **g** IL-27Rα expression on *Runx3d*^–/–^ and LMC DP IELs. Bar graphs are a summary of 2 independent experiments with a total of 3 *Runx3d*^–/–^ and 3 LMC mice. **h** Intracellular staining for T-bet and ThPOK in CD4^+^CD8αα^+^ IELs of *Runx3d*^–/–^ and LMC mice. Histograms are representative of 3 independent experiments. **i** IL-10 expression after PMA and ionomycin stimulation of 3-day anti-CD3/CD28-stimulated CD4^+^CD8αα^+^ SI IELs from *Runx3d*^–/–^ and LMC mice. The results are representative of 3 independent experiments. **j** CD4 versus CD8α profiles of CD4^+^CD8β^–^ SI IEL αβ T cells (top) and the frequency and number of CD4^+^CD8αα^+^ IELs (bottom) in *Runx3d*^–/–^ and LMC mice. The results show a summary of 7 independent experiments with a total of 8 *Runx3d*^–/–^ and 8 LMC mice. **k** Intracellular Bcl-2 protein contents in CD4^+^CD8αα^+^ IELs of *Runx3d*^–/–^ and LMC mice. The data are shown as histograms, and the bar graph summarizes 3 independent experiments with a total of 5 *Runx3d*^–/–^ and 6 LMC mice. **l** EdU incorporation was assessed in CD4^+^CD8αα^+^ IELs 16 h after EdU i.p. injection in *Runx3d*^–/–^ and LMC mice. The contour plot is representative of 4 independent experiments. **m** CD4 versus CD8α profiles of CD4^+^CD8β^–^ SI IEL αβ T cells (left) and the frequency and number of CD4^+^CD8αα^+^ IELs (right) in Bcl-2^Tg^ and WT mice. The results show a summary of 3 independent experiments with a total of 5 Bcl-2^Tg^ and 5 WT mice.

On another note, we were surprised that only in vitro activated but not freshly isolated DP IELs produced IL-10, as visualized using *Il10*-GFP reporter mice (Fig. 1b). Thus, IL-10 production is an inducible rather than a constitutive feature in DP IELs. The lack of steady-state IL-10 expression in DP IEL T cells contrasted with Foxp3^+^ Treg cells which constitutively produced IL-10 (Fig. 1c; Fig. S1b). Hence, we considered it unlikely that IL-10 production is the major mechanism by which DP IELs maintain immune quiescence under steady-state conditions.

To gain further insight into the immunoinhibitory function of DP IELs, we next examined the expression of molecules that are conventionally associated with immune suppression. We did not observe any significant expression of GITR, CD39, and CTLA-4 on CD4^+^CD8αα^+^ DP IELs (Fig. S3a) [9], and there was a complete lack of Foxp3^+^ cells in this population (Fig. S3b). Curiously, however, we found LAG-3 to be highly induced on DP IELs compared to conventional SI IEL CD4 T cells or Foxp3^+^ Treg cells (Fig. 1d). LAG-3 binds to MHC-II with high affinity and is a major mediator of immune suppression by Foxp3^+^ Treg cells [10]. Our observation that DP IELs express even larger amounts of LAG-3 than Foxp3^+^ Treg cells prompted us to reassess the regulatory pathways by DP IELs.

Notably, LAG-3 was highly expressed on CD4^+^CD8αα^+^ and CD8αα^+^ IEL T cells but not on CD4^+^ conventional T cells (Fig. 1d), prompting us to consider a molecular pathway where LAG-3 expression is associated with losing CD4 lineage identity but acquiring CD8αα^+^ lineage-associated characteristics. In this regard, CD4^+^CD8αα^+^ DP IELs had been proposed to differentiate from CD4^+^ T cells upon induction of the CD8 lineage-specifying transcription factor distal Runx3 (Runx3d) [11, 12], which we confirmed using *Runx3d*-YFP reporter mice (Fig. 1e) [13]. Thus, we next asked whether Runx3d would be associated with the LAG-3 induction in CD4^+^CD8αα^+^ DP IELs. If such were the case, we expected that *Runx3d*-deficiency would impair LAG-3 expression. To this end, we assessed LAG-3 expression on DP IELs of wild-type LMC and *Runx3d*-deficient (*Runx3d*^–/–^) mice. Indeed, LAG-3 levels were significantly reduced in *Runx3d*^–/–^ DP and CD8αα IELs (Fig. 1f; Fig. S4a), revealing a previously unappreciated role for Runx3d in promoting LAG-3 expression. On the other hand, the expression of other surface markers such as PD-1, CXCR3, CCR7, and CD44 were unaffected (Fig. S4c). In addition, *Runx3d*^–/–^ DP IELs contained significantly reduced amounts of granzyme B (Fig. S4b), indicating that Runx3d is necessary to fully establish a cytotoxic program in DP IELs.

Moreover, such a loss of expression was a direct effect of Runx3d on LAG-3 upregulation and not by interfering with the expression of IL-27Rα, the proprietary receptor for the cytokine IL-27 that promotes LAG-3 expression [10], because we found that IL-27Rα expression remained intact in the same cells (Fig. 1g; Fig. S4d, e). In fact, the abundance of IL-27Rα substantially increased in the absence of Runx3d (Fig. 1g). It would be interesting to examine whether IL-27Rα is a direct target of Runx3d, whereby a requirement for IL-27Rα in CD4^+^CD8αα^+^ DP IEL generation remains questionable because we did not observe a decrease in DP IEL frequencies and numbers in IL-27Rα-deficient mice (Fig. S5).

To further determine how *Runx3d*-deficient DP IELs differ from their WT counterparts, we next assessed the expression of transcription factors associated with DP IEL differentiation. While T-bet is expressed, ThPOK is mostly absent in CD4^+^CD8αα^+^ DP IELs [14], and we documented that this was also the case for *Runx3d*^–/–^ DP IELs (Fig. 1h). On the other hand, we found that IL-10 production by activated DP IELs was substantially decreased in *Runx3d*^–/–^ mice (Fig. 1i; Fig. S6a) as was also the case of IL-17a expression (Fig. S6b). Thus, together with the loss of LAG-3 expression, these results show that Runx3d is not required for their generation but necessary to promote and fully establish the immunoregulatory function of CD4^+^CD8αα^+^ IELs.

As an unexpected consequence of *Runx3d*-deficiency, we noticed that the abundance of DP IELs dramatically increased in the absence of Runx3d (Fig. S7a). While the frequency and number of CD4^+^ IELs were unaffected (Fig. S7b), *Runx3d*-deficiency resulted in a dramatic increase in CD4^+^CD8αα^+^ DP IEL frequencies and numbers (Fig. 1j), but without having detrimental effects on CD4 T cells in the thymus and spleen (Fig. S7c). In addition, the number of CD8αα^+^ IELs also remained unaffected (Fig. S7d), and we did not find any change in the Foxp3^+^ CD4 T cell population either (Fig. S8). Altogether, these results identify Runx3d as a negative regulator of CD4^+^CD8αα^+^ DP IELs.

Mechanistically, the increase in DP IEL cell numbers could be explained by either increased cell survival or increased cell proliferation, without necessarily excluding a combined effect. Here, we found that *Runx3d*^–/–^ DP IELs contained significantly increased amounts of the anti-apoptotic factor Bcl-2 compared to WT mice (Fig. 1k), indicating that increased cell survival contributes to the increased abundance of DP IELs. On the other hand, the proliferation of *Runx3d*^–/–^ DP IELs did not significantly differ from that of WT DP IELs, as assessed by in vivo EdU incorporation assays (Fig. 1l; Fig. S9a). Therefore, the increased number of CD4^+^CD8αα^+^ IELs can be mostly attributed to their increased cell survival rather than cell proliferation. Indeed, *Runx3d*^–/–^ DP IELs contained significantly lower amounts of active caspase-3 than their WT counterparts (Fig. S9b), indicating improved cell survival in the absence of Runx3d.

If such were the case, we expected that the forced expression of Bcl-2 would expand the size of the CD4^+^CD8αα^+^ SI IEL pool, which we then confirmed in Bcl-2 transgenic mice (Bcl-2^Tg^). Compared to wild-type controls, Bcl-2^Tg^ mice contained a substantially increased numbers of DP T cells in the SI epithelium (Fig. 1m), but without affecting the number of CD4^+^ IELs (Fig. S10). Thus, the DP IEL compartment is primarily controlled by limiting their cell survival, and Runx3d is a major player in this process.

Collectively, our results identify Runx3d expression as a new regulatory mechanism that constrains the size of the SI CD4^+^CD8αα^+^ DP IEL pool but supports the expression of LAG-3 and induction of IL-10. These findings further put forward a new transcriptional circuitry of DP IEL differentiation and phenotype acquisition, while it remains to be assessed what cellular signals drive and maintain this Runx3d-mediated pathway in CD4^+^CD8αα^+^ IELs.

## Supplementary information

Suppl Figures and Methods

## Data Availability

All data are available in the main text or the supplementary materials.
