# Supplementary material for: Runx3d controls the abundance and functional differentiation of CD4+CD8αα+ intraepithelial T cells
Source: Cell Death Discov. 2023 Apr 12;9:123. doi: 10.1038/s41420-023-01415-z (PMC10097811; doi:10.1038/s41420-023-01415-z)
Supplement: Supplementary file 1 — Suppl Figures and Methods [file 41420_2023_1415_MOESM1_ESM.pdf]

Supplementary Figure 1

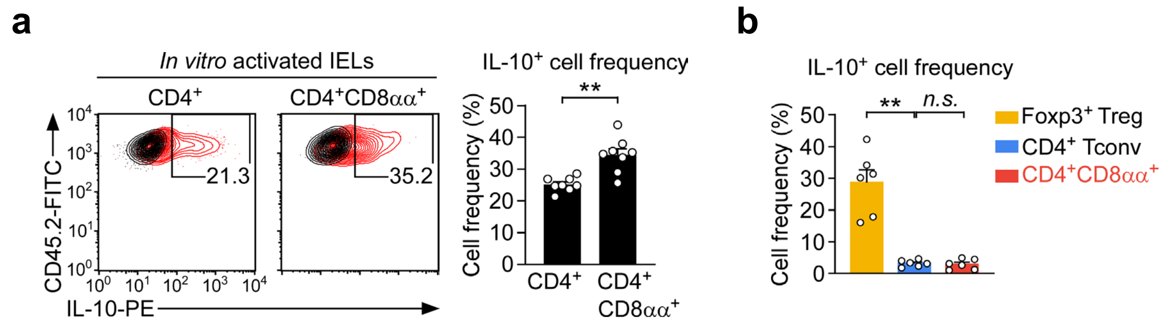

Supplementary Figure 1. IL-10 expression in CD4⁺CD8αα⁺ IELs

a. IL-10 expression was assessed in *in vitro* activated CD4⁺CD8αα⁺ SI IELs after 4 hours of PMA and ionomycin stimulation in the presence of brefeldin A. IL-10 expression (red) is overlaid on isotype control antibody staining (black). Contour plots are representative, and bar graphs are summary of 4 independent experiments with a total of 8 C57BL/6 mice.

b. Frequency of IL-10⁺ cells among the indicated SI IEL populations of *Foxp3*-GFP reporter mice. Results are a summary of 3 independent experiments with a total of 6 *Foxp3*-GFP reporter mice.

Supplementary Figure 2

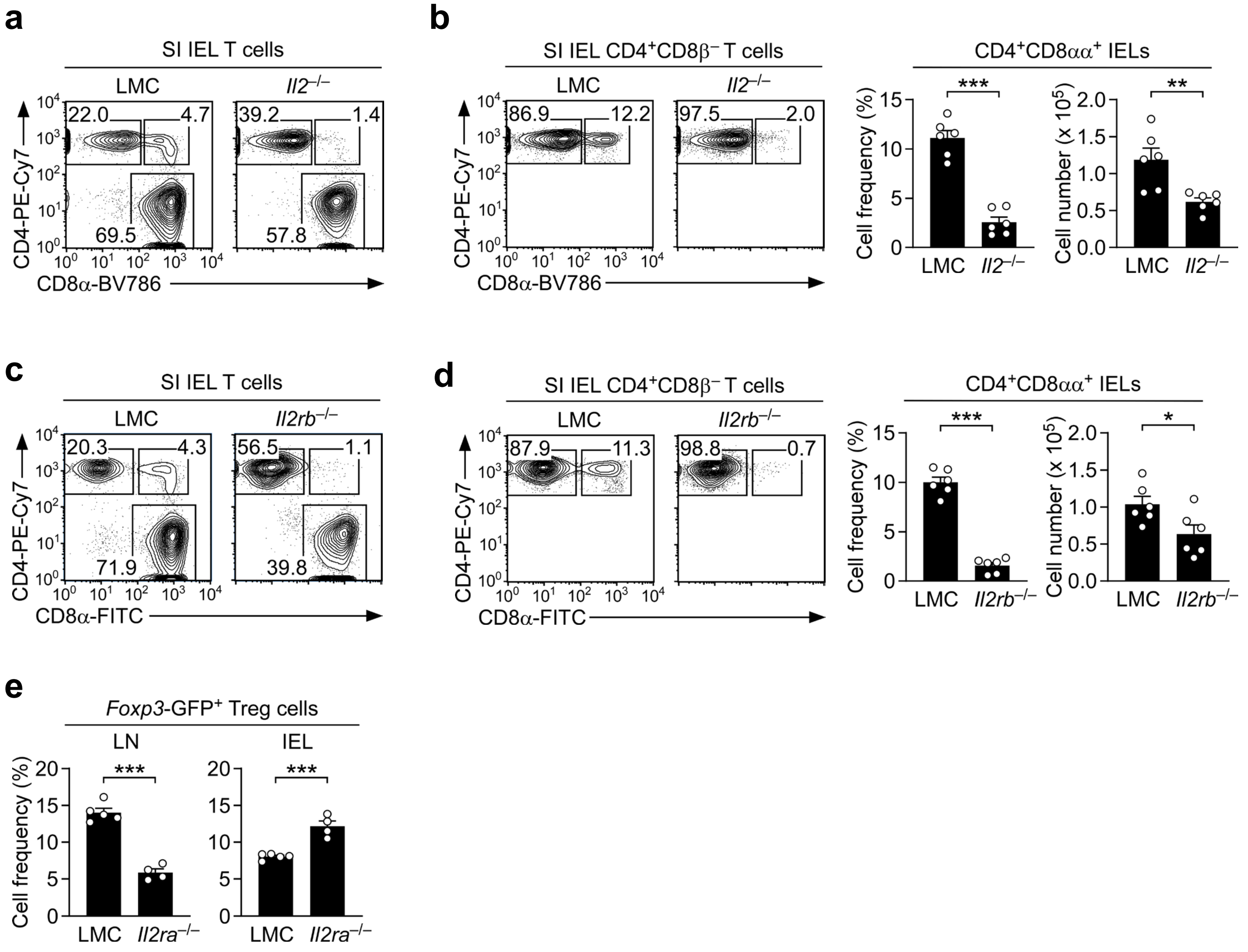

Supplementary Figure 2. SI IEL T cells in IL-2- and IL-2Rβ-deficient mice

a. CD4 versus CD8α profiles of SI IEL T cells in *Il2*$^{-/-}$ and littermate control (LMC) mice. Results are representative of 3 independent experiments with a total of 6 *Il2*$^{-/-}$ and 6 LMC mice.

b. CD4 versus CD8α profiles of CD4$^+$CD8β^- SI IEL T cells (left), and the frequency and number of CD4$^+$CD8$\alpha\alpha^+$ IELs (right) in *Il2*$^{-/-}$ and LMC mice. Results are representative of 3 independent experiments with a total of 6 *Il2*$^{-/-}$ and 6 LMC mice.

c. CD4 versus CD8α profiles of SI IEL T cells in *Il2rb*$^{-/-}$ and LMC mice. Results are representative of 3 independent experiments with a total of 6 *Il2rb*$^{-/-}$ and 6 LMC mice.

d. CD4 versus CD8α profiles of CD4$^+$CD8β^- SI IEL T cells (left) and the frequency and number of CD4$^+$CD8$\alpha\alpha^+$ IELs (right) in *Il2rb*$^{-/-}$ and LMC mice. Results are representative of 3 independent experiments with a total of 6 *Il2rb*$^{-/-}$ and 6 LMC mice.

e. *Foxp3*-GFP$^+$ cell frequencies among CD4$^+$ LNs and SI IELs of LMC and *Il2ra*$^{-/-}$*Foxp*3-GFP reporter mice. Bar graphs show summary of 2 independent experiments with a total of 4 *Il2ra*$^{-/-}$*Foxp*3-GFP reporter and 5 LMC mice.

Supplementary Figure 3

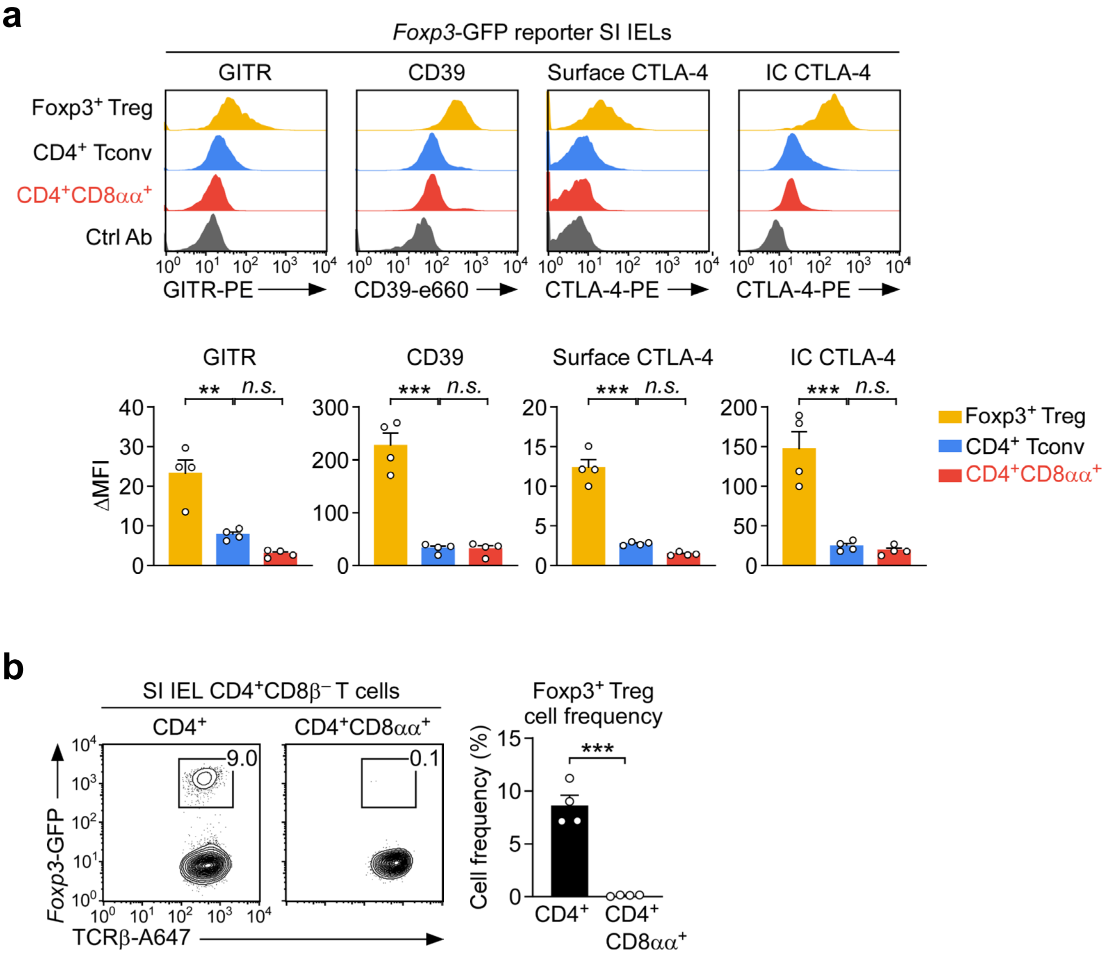

Supplementary Figure 3. Immunosuppressive phenotypes of CD4⁺CD8αα⁺ IEL T cells

a. Indicated subsets of SI IEL αβ T cells were assessed for expression of GITR, CD39, and both surface and intracellular (IC) CTLA-4. Histograms (top) are representative, and bar graphs (bottom) show summary of 2 independent experiments with a total of 4 *Foxp*3-GFP reporter mice.

b. *Foxp3*-GFP⁺ cell frequencies among CD4⁺ and CD4⁺CD8αα⁺ SI IELs of *Foxp*3-GFP reporter mice. Contour plots are representative (left), and bar graphs are summary of 3 independent experiments with a total of 4 *Foxp*3-GFP reporter mice (right).

Supplementary Figure 4

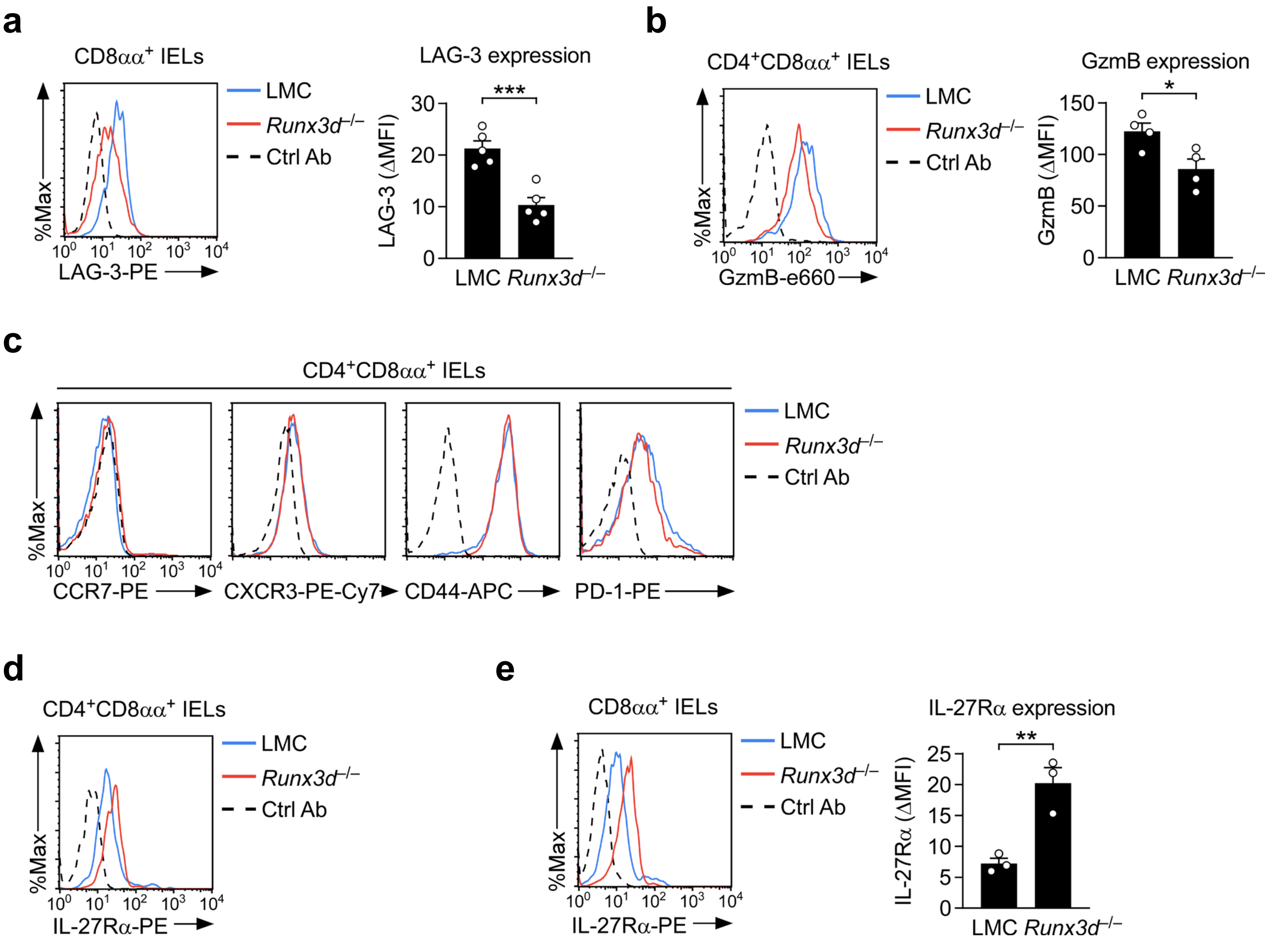

Supplementary Figure 4. Phenotypic characterization of SI IEL T cells in *Runx3d*$^{-/-}$ mice

a. LAG-3 expression was assessed on SI CD8αα$^+$ IELs of *Runx3d*$^{-/-}$ and LMC mice. Histograms are representative and bar graphs show summary of 4 independent experiments with a total of 5 *Runx3d*$^{-/-}$ and 5 LMC mice.

b. Intracellular granzyme B expression was assessed in fixed and permeabilized CD4$^+$CD8αα$^+$ SI IEL αβ T cells of *Runx3d*$^{-/-}$ and LMC mice. Histogram is representative (left) and bar graph (right) shows summary of a total of 4 *Runx3d*$^{-/-}$ and 4 LMC mice.

c. Surface expression of the indicated surface markers on CD4$^+$CD8αα$^+$ SI IEL αβ T cells of *Runx3d*$^{-/-}$ and LMC mice. Results are representative of 2 independent experiments with a total of 6 *Runx3d*$^{-/-}$ and 6 LMC mice.

d. IL-27Rα expression was assessed on SI CD4$^+$CD8αα$^+$ IELs of *Runx3d*$^{-/-}$ and LMC mice. Histograms are representative of 2 independent experiments with a total of 3 *Runx3d*$^{-/-}$ and 3 LMC mice.

e. IL-27Rα expression was assessed on SI CD8αα$^+$ IELs of *Runx3d*$^{-/-}$ and LMC mice. Histograms are representative (left) and bar graph (right) show summary of 2 independent experiments with a total of 3 *Runx3d*$^{-/-}$ and 3 LMC mice.

Supplementary Figure 5

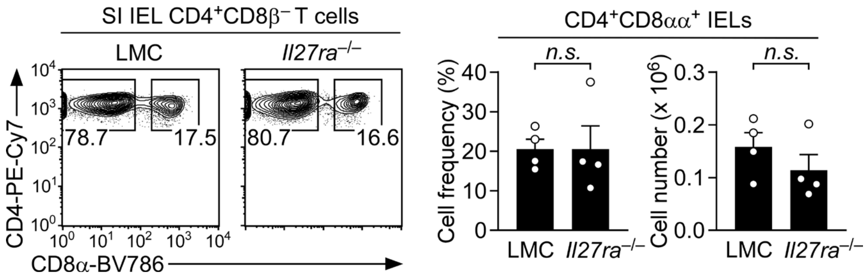

Supplementary Figure 5. CD4⁺CD8αα⁺ SI IEL αβ T cells of *Il27ra*–/– mice
Frequency and number of CD4⁺CD8αα⁺ SI IEL αβ T cells in *Il27ra*–/– and LMC mice. Contour plot is representative (left), and bar graphs (right) show summary of a total of 4 *Il27ra*–/– and 4 LMC mice.

Supplementary Figure 6

a

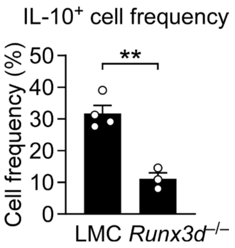

b

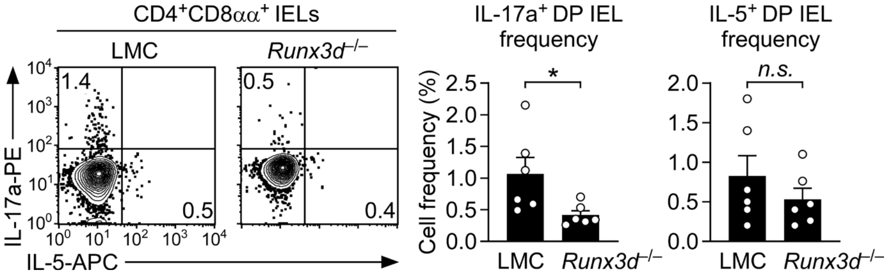

Supplementary Figure 6. Cytokines production of $CD4^+CD8\alpha\alpha^+$ SI IEL T cells in *Runx3d*$^{-/-}$ mice

a. Frequency of IL-10^+ IELs upon 4 hours of PMA and ionomycin stimulation of *in vitro* activated $CD4^+CD8\alpha\alpha^+$ SI IELs from *Runx3d*$^{-/-}$ and LMC mice. Results are the summary of 3 independent experiments with a total of 3 *Runx3d*$^{-/-}$ and 4 LMC mice.

b. Frequency of IL-$17a^+$ and IL-5^+ IELs upon 4 hours of PMA and ionomycin stimulation of freshly isolated $CD4^+CD8\alpha\alpha^+$ DP IEL $\alpha\beta$ T cells from *Runx3d*$^{-/-}$ and LMC mice. Contour plot is representative (left), and bar graphs (right) show summary of a total of 6 *Runx3d*$^{-/-}$ and 6 LMC mice.

Supplementary Figure 7

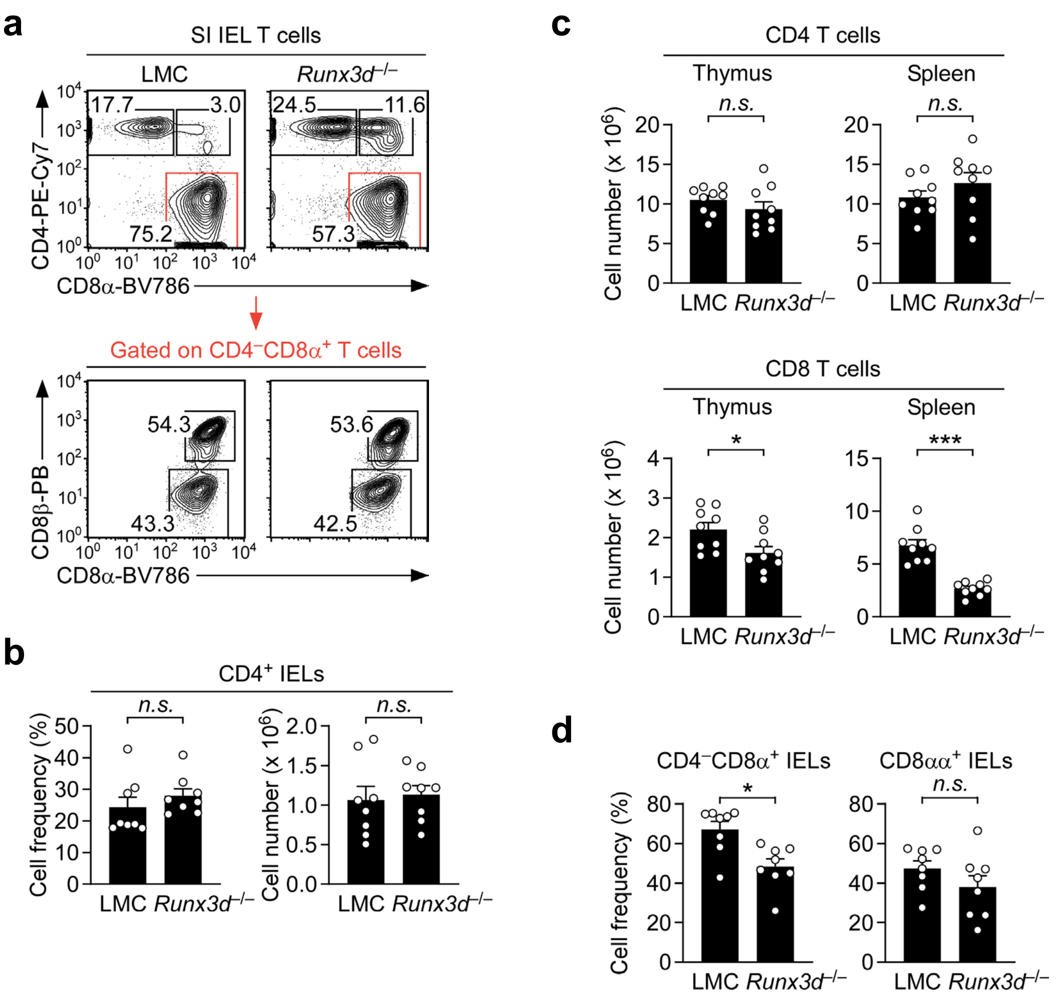

Supplementary Figure 7. T cell numbers and frequencies in *Runx3d*$^{-/-}$ mice

a. CD4 versus CD8 profiles of total SI IEL αβ T cells (top), and CD4^{-}CD8α^{+} SI IEL T cells (bottom) of *Runx3d*$^{-/-}$ and LMC mice. Results are representative of 7 independent experiments with a total of 8 *Runx3d*$^{-/-}$ and 8 LMC mice.

b. Frequency and number of CD4^{+} (TCRβ^{+}CD4^{+}CD8α^{-}) SI IEL T cells in *Runx3d*$^{-/-}$ and LMC mice. Results are the summary of 7 independent experiments with a total of 8 *Runx3d*$^{-/-}$ and 8 LMC mice.

c. Bar graphs show CD4 and CD8 T cell numbers in the thymus and spleen of *Runx3d*$^{-/-}$ and LMC mice. Results are summary of a total of 9 *Runx3d*$^{-/-}$ and 9 LMC mice.

d. Frequencies of CD4^{-}CD8α^{+} and CD8αα^{+} SI IEL T cells in *Runx3d*$^{-/-}$ and LMC mice. Results are the summary of 7 independent experiments with a total of 8 *Runx3d*$^{-/-}$ and 8 LMC mice.

Supplementary Figure 8

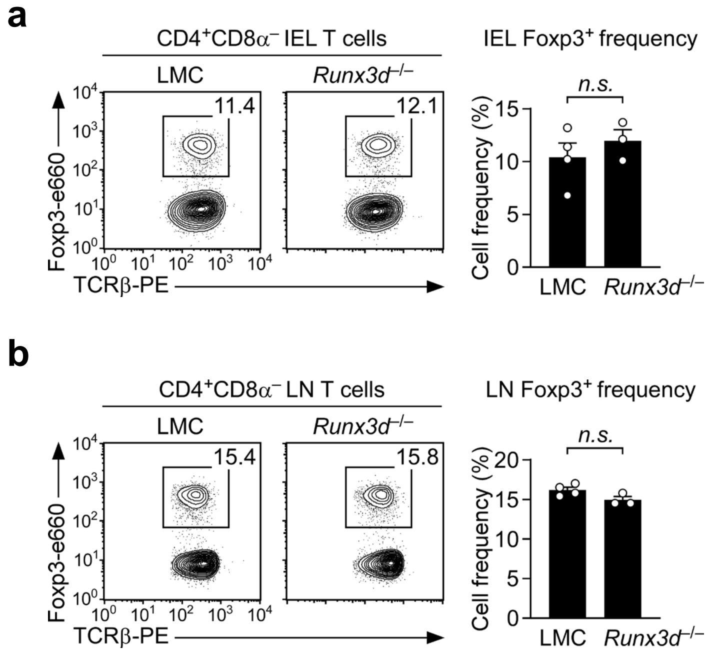

Supplementary Figure 8. Foxp3 expression in CD4⁺CD8α⁻ T cells of *Runx3d*$^{-/-}$ mice
a, b. Frequency of Foxp3⁺ cells among CD4⁺CD8α⁻ SI IEL αβ T cells (a) and CD4⁺CD8α⁻ LN αβ T cells (b) of *Runx3d*$^{-/-}$ and LMC mice. Contour plots are representative (left) and bar graphs (right) show summary of a total of 3 *Runx3d*$^{-/-}$ and 4 LMC mice.

Supplementary Figure 9

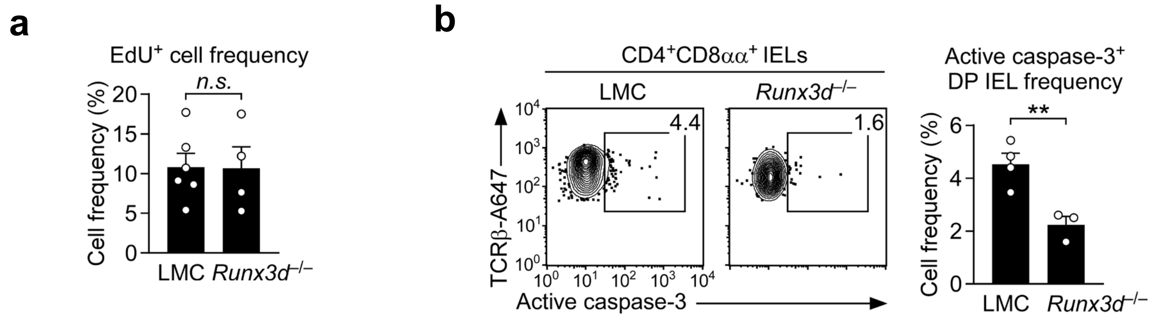

Supplementary Figure 9. Assessing cell proliferation and survival of DP IELs in *Runx3d*$^{-/-}$ mice

a. EdU incorporation in $CD4^+CD8\alpha\alpha^+$ SI IELs from *Runx3d*$^{-/-}$ and LMC mice. Results are the summary of 4 independent experiments with a total of 4 *Runx3d*$^{-/-}$ and 6 LMC mice.

b. Intracellular abundance of active caspase-3 was assessed in $CD4^+CD8\alpha\alpha^+$ SI IEL $\alpha\beta$ T cells of *Runx3d*$^{-/-}$ and LMC mice. Contour plot is representative (left) and bar graph (right) shows summary of a total of 3 *Runx3d*$^{-/-}$ and 4 LMC mice.

Supplementary Figure 10

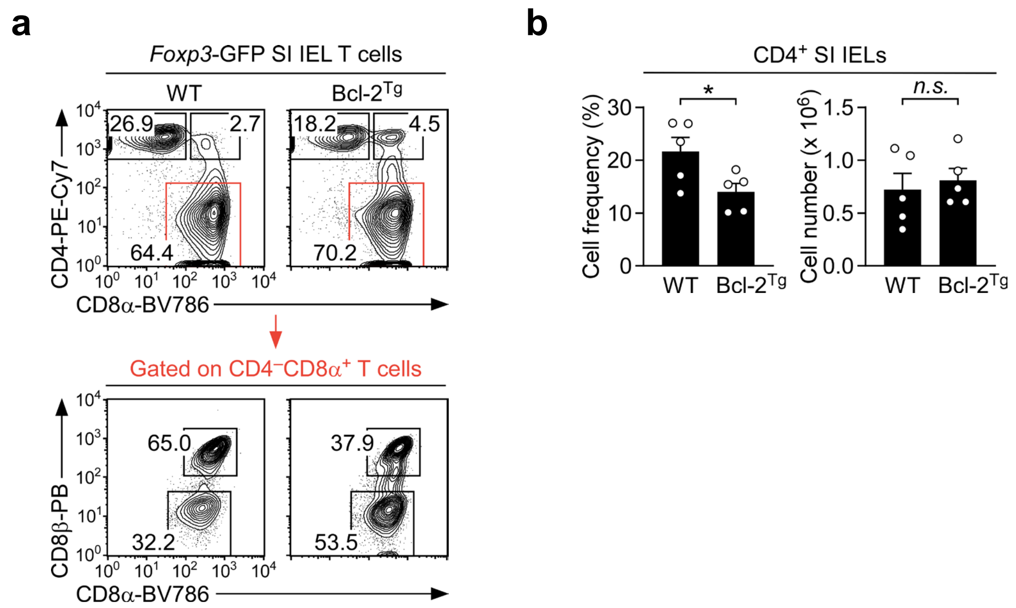

Supplementary Figure 10. SI IEL T cells in Bcl-2-transgenic mice

a. CD4 versus CD8 profiles of total SI IEL αβ T cells (top), and CD4⁻CD8α⁺ SI IEL αβ T cells (bottom) of Bcl-2^{Tg} and WT mice with *Foxp3*-GFP background. Results are representative of 3 independent experiments with a total of 5 Bcl-2^{Tg} and 5 WT mice.

b. Frequency and number of CD4⁺ (TCRβ⁺CD4⁺CD8α⁻) SI IEL T cells in Bcl-2^{Tg} and WT mice with *Foxp3*-GFP background. Results are the summary of 3 independent experiments with a total of 5 Bcl-2^{Tg} and 5 WT mice.

Supplementary Materials and Methods

Mice

C57BL/6NCrl (C57BL/6) mice were purchased from the Charles River Laboratories. *Foxp3*-GFP knock-in reporter mice were previously described and kindly provided by Dr. Vijay Kuchroo (Harvard University) [1]. *Il2*$^{-/-}$ mice [2], *Il2rb*$^{-/-}$ mice [3], *Il27ra*$^{-/-}$ mice [4], *Runx3d*-YFP reporter (*Runx3d*$^{+/-}$) mice [5], and *Il10*-GFP reporter mice [6] were previously reported and obtained from the Jackson Laboratory. Bcl-2 transgenic mice (Bcl-2^{Tg}) mice were previously reported [7] and bred into *Foxp3*-GFP knock-in reporter background. *Il2ra*$^{-/-}$ mice [8] were obtained from the Jackson Laboratory and crossed with *Foxp3*-GFP knock-in reporter mice. Animal experiments were performed using 5- to 12-week-old mice of both sexes. All animal experiments were reviewed and approved by the NCI Animal Care and Use Committee, and all mice were cared for in accordance with NIH guidelines.

Preparation of single-cell suspension

Single-cell suspension from thymus, lymph nodes (LNs), spleen [9] or small intestine intraepithelial cells (SI IELs) [10] were isolated as previously described. Briefly, thymus, LNs and spleen were pressed into a cell emulsion and pipetted into single-cell suspensions by passing through 100 μm nylon filters. SI IELs were eluted from the SI and recovered by negative selection using anti-EpCAM antibody (clone G8.8) and BioMag anti-rat IgG magnetic beads (Qiagen).

Flow cytometry

Single-cell suspensions were stained with fluorescence-conjugated antibodies with the following specificities: TCRβ (H57-597), CD45 (30-F11), LAG-3 (C9B7W), CD45.2 (104), IL-5 (TRFK5), CXCR3 (CXCR3-173), CD44 (IM7) and IL-27Rα (W16125D) from BioLegend; CD8β (eBioH35-17.2), GITR (DTA-1), CD39 (eBio1D3), IL-10 (JES5-16E3), ThPOK (T43-94), CCR7 (4B12), Granzyme B (NGZB), Foxp3 (FJK-16s) and Rat IgG2b kappa Isotype Control (eB149/10H5), IgG1κ isotype (P3.6.2.8.1) and T-bet (eBio4B10 (4B10)) from eBioscience; CD8α (53-6-7), IL-17a (TC11-18H10), PD-1 (J43), Hamster Anti-Mouse Bcl-2 set (3F11) and CD16/32 (2.4G2) from BD Biosciences (San Jose, CA); CD4 (GK1.5), CTLA-4 (UC10-4F10-11) and Ghost dye Violet 510 viability dye from Tonbo Biosciences. Foxp3, ThPOK, and T-bet intranuclear staining was performed using a Foxp3 intracellular staining buffer set (eBioscience), whereas CTLA-4, IL-10, IL-5, IL-17a, Granzyme B and Bcl-2 intracellular staining was performed using IC fixation kit (eBioscience), according to the manufacturer's instructions. IL-10 production in freshly isolated or *in vitro* activated SI IELs, and IL-5 and IL-17a in freshly isolated SI IELs were assessed by stimulation with of 50 ng/mL PMA and 1 μM ionomycin (Sigma-Aldrich) in the presence of 3 μg/mL brefeldin A (Invitrogen), as previously described [11].

For EdU detection, mice were intraperitoneally injected with EdU (1 mg per mouse). After 16 hours, IELs were harvested and EdU$^+$ cells were detected using the Click-iT Plus EdU Flow Cytometry Assay (Thermo Fisher Scientific).

For active caspase-3 assays, IELs were harvested and cultured in 37°C with serum-free RPMI 1640 medium. After 1 hour, cells were stained with Red-DEVD-FMK (Cleaved Caspase-3 Staining Kit (Red), Abcam) for 45 min in 37°C, and followed by the staining of surface markers and Ghost Violet 510 viability dye in 4°C.

Samples were acquired using LSRII or LSR Fortessa flow cytometers (BD Bioscience). Flow cytometry data were analyzed using the FlowJo v10.6.2 software.

In vitro activation of SI IELs

Freshly isolated SI IELs were cultured with 1 µg/mL plate-bound anti-CD3 (2C11; BioLegend) and anti-CD28 antibodies (37.51; BioLegend), plus 5 ng/mL TGF-β (R&D Systems) and 100 U/mL IL-2 (R&D Systems) for 3 days at 37°C. After *in vitro* activation, cells were harvested and assessed for IL-10 production, as described above.

Statistics and data presentation

All data sets were tested for normality using the Shapiro-Wilk normality test. Where data were normally distributed, statistical significance was assessed by paired or unpaired two-tailed Student's *t*-test, or one-way ANOVA with Tukey's multiple comparison correction using GraphPad Prism 8 software (GraphPad). Data are shown as mean ± SEM, and *P* values of less than 0.05 were considered significant. * $P < 0.05$, ** $P < 0.01$, *** $P < 0.001$, *n.s.*, not significant. All figures were generated by the software program Canvas X (Canvas GFX).

References

1. Bettelli E, Carrier Y, Gao W, Korn T, Strom TB, Oukka M, et al. Reciprocal developmental pathways for the generation of pathogenic effector TH17 and regulatory T cells. Nature. 2006;441:235-238.

2. Sadlack B, Merz H, Schorle H, Schimpl A, Feller AC, Horak I. Ulcerative colitis-like disease in mice with a disrupted interleukin-2 gene. Cell. 1993;75:253-261.

3. Chinen T, Kannan AK, Levine AG, Fan X, Klein U, Zheng Y, et al. An essential role for the IL-2 receptor in Treg cell function. Nat Immunol. 2016;17:1322-1333.

4. Yoshida H, Hamano S, Senaldi G, Covey T, Faggioni R, Mu S, et al. WSX-1 is required for the initiation of Th1 responses and resistance to L. major infection. Immunity. 2001;15:569-578.

5. Egawa T, Littman DR. ThPOK acts late in specification of the helper T cell lineage and suppresses Runx-mediated commitment to the cytotoxic T cell lineage. Nat Immunol. 2008;9:1131-1139.

6. Kamanaka M, Kim ST, Wan YY, Sutterwala FS, Lara-Tejero M, Galan JE, et al. Expression of interleukin-10 in intestinal lymphocytes detected by an interleukin-10 reporter knockin tiger mouse. Immunity. 2006;25:941-952.

7. Strasser A, Harris AW, Cory S. bcl-2 transgene inhibits T cell death and perturbs thymic self-censorship. Cell. 1991;67:889-899.

8. Willerford DM, Chen J, Ferry JA, Davidson L, Ma A, Alt FW. Interleukin-2 receptor alpha chain regulates the size and content of the peripheral lymphoid compartment. Immunity. 1995;3:521-530.

9. Li C, Park JH. Assessing IL-2-Induced STAT5 Phosphorylation in Fixed, Permeabilized Foxp3(+) Treg Cells by Multiparameter Flow Cytometry. STAR Protoc. 2020;1:100195.

10. Prakhar P, Gonzalez V, Park JH. High-yield enrichment of mouse small intestine intraepithelial lymphocytes by immunomagnetic depletion of EpCAM(+) cells. STAR Protoc. 2022;3:101207.

11. Prakhar P, Alvarez-DelValle J, Keller H, Crossman A, Tai X, Park YK, et al. The small intestine epithelium exempts Foxp3+ Tregs from their IL-2 requirement for homeostasis and effector function. Jci Insight. 2021;6:(21):e149656.
